# Gamblers’ Perception of the Playscan Risk Assessment: A Mixed-Methods Study

**DOI:** 10.1007/s10899-021-10043-0

**Published:** 2021-06-05

**Authors:** David Forsström, Alexander Rozental, Emma Wiklund, Per Carlbring, Philip Lindner

**Affiliations:** 1 Centre for Psychiatry Research, Department of Clinical Neuroscience, Karolinska Institutet and Stockholm Health Care Services, Region Stockholm, Stockholm, Sweden; 2 Department of Psychology, Stockholm University, Stockholm, Sweden; 3 UCL Great Ormond Street Institute of Child Health, London, UK; 4 Centre for Dependency Disorders, Stockholm Health Care Services, Region Stockholm, Stockholm, Sweden; 5 The Centre for Psychotherapy, Education and Research, Liljeholmstorget 7, SE-117 63 Stockholm, Sweden

**Keywords:** Responsible gambling, Risk assessment, Playscan, Negative attitude, High-risk gamblers

## Abstract

Responsible gambling (RG) tools are globally widespread; they aim to prevent or decrease the harm caused by gambling. However, existing research suggests that several included features do not decrease gambling or significantly reduce the subsequent harm. Most of the previous studies have used gambling data to understand the changes in gambling behavior. However, the literature lacks research regarding gamblers’ experience and perception of RG tools, which may provide insight into increasing the usage and effectiveness of RG tools. This mixed-methods study aimed to explore gamblers’ perception of their risk assessment in the RG tool Playscan regarding developing harmful gambling problems. Overall, 757 participants rated the perceived accuracy of their risk assessment and their perception of the overall RG tool that conducted the assessment. Participants were also allowed to leave a comment providing feedback, which was analyzed using thematic analysis. Quantitative data was analyzed using logistic regression and structural equation modeling. Qualitative analyses revealed that most of the participants were pleased with the risk assessment and found it helpful. Moderated mediation analysis showed that participants’ assessment agreement partially mediated the association between expressing a negative view and their general view of Playscan. These results highlight the need to decrease the level of disagreement for promoting a better general view of RG tools to potentially increase their usage and effectiveness.

## Background

Online gambling has become the most prevalent gambling type in several countries; however, it comes with a unique set of risks—for developing gambling problems—as well as opportunities—for prevention and intervention. The so-called responsible gambling (RG) tools refer to various measures, along the voluntary-obligatory and discrete-invasive spectrums, which aim to prevent or mitigate the harm of online gambling (Blaszczynski et al., 2004). Despite their wide implementation and need in many jurisdictions, there is a lack of research examining the effectiveness of these measures (Forsström et al., 2020c; McMahon et al., 2019); moreover, several of these measures seem to have been implemented, without any scientific evidence, or they have been generalized from the measures developed for another jurisdiction (Williams et al., 2012). Existing studies indicate that gambling companies typically comply with RG legislation and practices (Forsström & Cisneros Örnberg, 2019; Marionneau & Järvinen-Tassopoulos, 2017); however, several measures are yet to be developed to adequately protect gamblers from potential risks. For example, Fiedler et al. (2020) found that companies did not intervene despite the discovery of risks.

Risk assessment, based on self-reports and gambling behavior-tracking is a popular feature of RG tools. A recent review on behavior tracking elucidated the lack of empirical research on this approach, which could be due to the limited accessibility of researchers, up until recently, to use the data collected routinely by gambling providers (Chagas & Gomes, 2017). Risk assessments are often based on the assumption of high correlations between the amount and intensity of gambling and the risk of developing gambling problems, as presented in the actual gambling data. Several studies have proposed different moderately successful strategies to determine risk using behavioral markers detected through the gambling data (Adami et al., 2013; Braverman & Shaffer, 2012; Dragicevic et al., 2011, 2015; Haeusler, 2016; Percy et al., 2016; Philander, 2014). Despite these behavioral markers, studies are yet to explore strategies which incorporate the gambler’s perspective and their value into risk assessment tools. Existing research on the user experience of RG tools may aid in developing tools with higher utility and effectiveness. It is particularly challenging to attract the right type of gamblers (those with at least some degree of gambling problems), to start and continue using these RG tools. An analysis conducted by Forsström et al. (2016) on the user data for the widespread and well-studied RG tool, Playscan, demonstrated that despite a high initial usage rate, the tool was used on a recurrent basis by very few users; additionally, despite the voluntary participation to use Playscan in this study, 7.9% of the participants did not use the tool at all (Forsström et al., 2016). Self-test was found to be the most widely used feature of Playscan (GamTest; Forsström et al., 2020a, b; Jonsson et al., 2017); it is used to determine the degree of negative consequences a user experiences, due to his or her gambling activities (Forsström et al., 2020a; Jonsson et al., 2017). Furthermore, the self-test is one of the most widely used features by the participants, thereby, serving as a gateway to using the rest of the tool (Forsström et al., 2017). Nonetheless, the frequency of repeated usage was low among participants, indicating that despite continued gambling, users gradually stopped assessing their risk level, and that few participants were aware of Playscan, even though they had signed up for using it (Forsström et al., 2017). Recently, Gainsbury et al. (2020) investigated general RG measures and reported similar findings, where participants found the tools provided to be relevant, and the usage of an RG measure was reported by a fairly lower percentage of participants (Gainsbury et al., 2020). Wood and Wohl (2015) reported that at-risk gamblers, who opted to use Playscan, subsequently engaged in deposits and wagers of smaller magnitude, over the next 24 weeks, compared to those in a propensity-matched comparison group. Similarly, Forsström et al., (2020b) found no decrease in net loss of participants, after using Playscan for a short period of time, while low-risk gamblers reported losing more money after using Playscan. However, Forsström et al. and’s. (2020b) findings should be generalized with caution due to several sample-related limitations and the low degree of repeated use of the tool reported by the participants (gamblers), which may explain the small observed effect of Playscan; although the attempts of the study to model the causal effect of usage per se using complier average causal estimation or other appropriate statistical techniques are noteworthy and well-suited for this field (i.e., large samples and objective measures of adherence). Studies have reported contradictory findings regarding the efficacy of Playscan in decreasing gambling, where one study indicates a significant decrease, while the other suggests no reduction (Forsström et al., 2020b; Wood & Wohl, 2015). In addition, Auer and Griffiths (2018) found that highlighting the discrepancy in the perceived gambling amount, by giving gamblers feedback in their study regarding the actual amount gambled, did not decrease future spending. Playscan users joined the tool out of curiosity, believing that using the tool could decrease their gambling, but they did not decrease their gambling as a consequence of using Playscan (Griffiths et al., 2009).

Studies that have investigated gamblers’ views on using RG measures are in most cases based on gamblers’ attitudes toward RG and not their actual usage of these measures. Thus, the existing research lacks an explanation for gamblers’ actual usage of RG measures and its impact on their gambling activities. A survey conducted across about 100 countries found that gamblers perceived RG measures as positive (Gainsbury et al., 2013). Moreover, positive attitudes toward RG measures were associated with gender (female), age (young), playing random games only, being a moderate-risk or problem gambler, reporting high impact from gambling advertisements as well as the personality traits agreeableness, openness, and neuroticism, and playing low risk games only. Reporting a high amount of spending on gambling and personality trait extraversion were inversely related to low risk (Engebø et al., 2019). Ivanova et al. (2019) revealed that individuals demonstrating a higher level of risk according to the Problem Gambling Severity Index (Ferris & Wynne, 2001), reported lower positivity toward RG measures; however, it was not disclosed whether these gamblers, included in the study, had used RG measures. Gainsbury et al. (2018) investigated the perception of different RG messages by different types of gamblers, and suggested that different types favored different messages. Thus, the findings indicate a need for customizing RG messages. This conclusion is consistent with several studies that have shown the effect of personalized vs normative feedback aimed at gamblers. Auer and Griffiths (2020) found that after receiving personalized feedback, gamblers wagered lower amounts for up to seven days. Other studies found similar effects suggesting that personalized and normative feedback impacted gambling among participants, compared to the control group (Auer & Griffiths, 2016; Auer et al., 2018). Therefore, there is a dearth of qualitative and quantitative research on gambling individuals’ perception of RG tools and, particularly, regarding their experience of receiving feedback on their risk assessment.

Thus, the current mixed-methods study aimed to investigate Norwegian online gamblers’ views of their risk assessment presented through Playscan. The following research questions were explored in this study:

1. What are the emerging themes when gamblers comment on the risk assessment received via Playscan?
2. How accurate is the risk assessment and what is the overall view of Playscan and how does that relate to the risk of developing gambling problems?

## Method

### Norsk Tipping

Norsk Tipping is a Norwegian-owned gambling provider, which monopolizes in the provision of gambling services—land-based and online—within the country. Since January 14, 2014, Norsk Tipping employs Playscan as an RG tool on its gambling website (www.norsk-tipping.no) and since March 19, 2015, Playscan usage has been mandated for Norsk Tipping’s online customers.

### “Playscan”—The Responsible Gambling Tool

Playscan aims to decrease the gambling activities of at-risk gamblers associated with the gambling sites that employ the tool. The background of this tool is based on two major concepts: (1) *Motivational interviewing* refers to a non-directive therapeutic style that focuses on helping individuals overcome their ambivalence toward change; it involves an important aspect of evoking the “change talk,” which refers to exploring the individual’s subjective desire, ability, and purpose of changing a particular behavior (e.g., cutting down on gambling), rather than providing them with the objective reasons to do so (Hettema et al., 2005; Miller & Rollnick, 2002). (2) *Stages of change* are characterized by a transtheoretical model describing change as an ongoing process: precontemplation, contemplation, preparation, action, and maintenance (Norcross et al., 2011; Prochaska et al., 1993). The model suggests that change involves specific stages and tasks that need to be experienced and resolved by an individual. Thus, the stages of change represent the ability of an individual to change a certain behavior, ranging from having no intention to change, maintaining progress, and avoiding relapse. Therefore, interventions aimed at helping an individual alter their behavior should be consequently tailored to match their current stage. Playscan comprises three components that aim to promote behavioral changes: (1) *risk assessment*, which is based on the users’ gambling history and different markers of excessive gambling, such as night owling—staying up late to continue gambling), chasing losses—trying to win back lost money, and the time and money spent on gambling. In addition, the risk assessment factors in the self-rated GamTest score outline different consequences of gambling. Some of the questions included in the questionnaire were similar to those in the Problem Gambling Severity Index (Ferris & Wynne, 2001). However, the GamTest also comprises several questions about the time and money spent on gambling. Gambling history and the results of the self-test both carry equal weightage in the overall risk assessment result. The assessment presents three different risk levels to indicate risk of developing gambling problems: green (low risk), yellow (moderate risk), and red (high risk). If there is no gambling data to base the assessment no risk rating will be provided. (2) *Feedback from the risk assessment* is communicated to the user via a messaging service built into the tool. Playscan is accessible via a web browser from the gambling site webpage; however, users need to log in to access the assessment, which is indicated via a notification message displayed while logging into the gambling website. Moreover, the user will not be assigned a risk level (i.e., color) by the assessment, if he or she has not gambled on the website, during the period for the risk assessment (one week). (3) The user can choose to *receive advice,* after receiving feedback from the assessment, on how to restrict his or her level of gambling, which includes different strategies for reducing gambling behavior, such as, budget-setting or taking a break from gambling entirely via self-exclusion; this feedback given is based on the results of the risk assessment. Additional information about the tool is available from Forsström et al. (2016) and on the Playscan website (www.playscan.com).

### Measures and Procedure

Data was collected from the Norsk Tipping website. Participants included in this study were only required to: have a Norsk Tipping gambling account and opt for using the RG tool, Playscan. The sample recruitment was not limited by exclusion criteria or sample size requirements. Users were given the opportunity to evaluate and comment on the results, after receiving their risk assessment. For this purpose, the gamblers who participated in their own risk analysis, received a pop-up notification, upon logging into Playscan via Norsk Tipping, asking them if they wanted to participate in an evaluation of Playscan and their recent risk assessment. Information on voluntary participation was also provided here, and those who volunteered and fulfilled the inclusion criteria were recruited in this study. Data were collected over approximately 50 days.

Participants’ (gamblers’) general evaluation of Playscan, and their agreement with the risk assessment received on a scale of green to red), was measured using a 9-point Likert-scale ranging from 1 (*do not agree at all*) and 9 (*totally agree*). Additionally, an open-ended question was added to the tool (“Other comments”), which encouraged the gamblers to comment on the risk assessment results in their own words.

### Participants

A total of 757 responses were received with comments. Of these, 161 (21.3%) belonged to women, while 596 (78.7%) belonged to men. Participants ranged between the age of 20 and 87 years (Mean [M] = 49.4 years, Standard Deviation [SD] = 13.1).

Both the evaluation ratings were compared between the genders. However, no significant difference were found between men and women in their evaluation ratings regarding the accuracy of their risk assessment (M_men_ = 4.87, SD = 3.44; M_women=_ 5.06, SD = 3.70; *t*(755) = − 0.615; p = 0.539), and for Playscan (M_men_ = 5.13, SD = 3.32; M_women_ = 5.61, SD = 3.60; *t*(755) = − 1.592; *p* = 0.112)002E

Similarly, chi^2^- test results revealed no significant differences in the distribution of risk ratings between men and women (N = 755; *χ*^*2*^[3] = 3.666; *p* = 0.300). The risk distribution of the sample was as follows: 34 (4.5%), 26 (4.4%), and 8 (5%) with no risk assessment; 342 (45.2%) 267 (44.8%), and 75 (46.6%) with a low-risk rating; 165 (21.8%), 124 (20.8%), and 41 (25.5%) with a medium-risk rating; and 216 (28.5%), 179 (30%), and 37 (23%) with a high-risk rating; among the overall sample, men, and women, respectively.

### Analysis

The free-text data, obtained from the open-ended question, was analyzed using thematic analysis, which was performed in accordance with the six steps established through previous research—familiarize yourself with your data, generate initial codes, search for themes, review the themes, define and name the themes, and produce the report (Braun & Clarke, 2006; Terry et al., 2017). Qualitative analysis was performed by one of the authors, who was a BSc student of psychology student at the time of the analysis with no conflict of interest. Furthermore, every comment was categorized into a primary theme to facilitate quantitative analysis based on the thematic structure. The final thematic structure and classifications were verified by the first author.

Thus, quantitative analyses were performed using the extracted themes. Participants who expressed neutral themes and/or received a no-risk assessment were excluded from these analyses; thus, the sample data of 552 participants were incorporated into the analysis. First, univariate associations were examined by performing ordinary or logistic regression models (the former with bias-corrected bootstrapped confidence intervals to account for skewed distribution). Second, a moderated mediation model (Muller et al., 2005) was performed within a structural equation modeling framework to examine the direct association between expressing a negative theme and an overall view of Playscan, and an indirect association via agreement with assessment. The moderating effects of the risk of developing gambling problems (binarized as either green or yellow/red) on each of the three individual paths were examined. Confidence intervals in the moderated mediation model were calculated using bias-corrected bootstrapping (k = 1000).

### Ethical Considerations

This study was approved by the Swedish Ethical Review Authority (Dnr. 2014/545–31 and 2020–02923) and was conducted in accordance with the Helsinki Declaration. All participants provided informed consent to have their anonymized data shared with researchers, upon initiating Playscan. Data was provided to the researchers with an anonymized study ID.

## Results

### Qualitative Results

As presented in Table 1, five themes emerged from the qualitative analysis of the overall responses: positive comments (294 responses; 39%), negative comments (284 responses; 37.5%), explanations for the risk assessment (131 responses; 17.3%), confusion about Playscan (19 responses; 2.5%), and technical issues (11 responses; 1.5%). Very few responses contributed to the last two themes, while 18 responses could not be categorized at all, due to ambiguity or incomplete responses.

**Table 1:** Themes based on the answers

Positive comments
Negative comments
Explanations for the risk assessment
Confusion about Playscan
Technical issues

#### Positive Comments

Positive comments were characterized by agreement between the gamblers’ received and perceived risk assessment regarding their gambling. Playscan was generally perceived as helpful, either for the player himself or for others. Participant responses ranged from "Okay" or "Good" to more detailed positive comments, such as: “*A good way to get statistics, and always good to see your own consumption.*”

Several responses expressed that Norsk Tipping acted responsibly by mandating gamblers to use Playscan. Gamblers stated that Playscan provides encouragement and utility for those who need it; however, they suggested that they themselves did not need the tool. Additionally, gamblers also expressed that the tool helped them overall; however, they did not provide a detailed explanation on how and what the tool helped them with. An example of such a statement was: “*thanks for monitoring the people and helping people that have a gambling problem….*”

#### Negative Comments

Negative comments were characterized by gamblers’ dissatisfaction or criticism of the risk assessment received and/or Playscan. The gamblers criticized the risk assessment, and questioned it by providing an explanation for why they were not at-risk gamblers. Several comments also pointed out that the risk assessment does not consider important factors, such as, multiple users for certain gambling accounts in a so-called company game, website price hikes, or individual income.

A sub-theme that emerged was gamblers’ irritation, which was conveyed through expressions and words associated with anger and frustration, such as, the phrase "Bare tull" meaning "only nonsense" was used 38 times. In addition to the risk assessment, gamblers also criticized the overall tool of Playscan. Many participants also felt that implementing Playscan was a bad idea, and it would not help those with gambling problems. Some respondents commented that they did not want to be monitored, and their gambling their own concern only. Moreover, several participants questioned the validity of the GamTest (self-test), and they believed that those with gambling problems would not answer the questions honestly. An example of a negative comment was: “*Bad model, assumes it does not take into account set limits and self-control*.”

#### Explanations for the Risk Assessment

Gamblers provided explanations for their risk assessment by commenting on the potential cause for receiving a certain result. For example, their economic background (high income, no loan) allowed them to gamble large sums of money or several other users shared their account, thus, falsely indicating that the account holder was at high risk. Some respondents also conveyed that the money, they have lost provides community support and serves good causes (the surplus from Norsk Tipping goes to different projects in sports and culture sector). Examples of this were: “*Playscan should not draw conclusions without knowing the facts! My card is used to deliver games for a* ‘*tipping team" consisting of a group of friends. When Playscan cannot compensate for such things, they should also not offer any kind of risk assessment to people!*’ and “*Playscan takes no account of personal income*.”

#### Confusion about Playscan

A minor theme emerged from expressing ambiguities regarding Playscan. Some gamblers could not understand the purpose of the tool and/or differentiate between Norsk Tipping and Playscan; some participants even commented on the things that concerned Norsk Tipping and not Playscan. An example was: “*My problem with gambling is that I encounter a cynical industry that steals my money. Is no worse than trying to get back what has been taken from me. Is it just me who is stupid and naive who thinks there is good in the world but lets me get robbed over and over?*’.

#### Technical Issues

Some of the comments outlined the technical problems with Norsk Tipping’s application and/or website, and even included questions about the different gambling services provided by Norsk Tipping. Despite their similarity, the main difference between this theme and previous one is the specificity of technical problems emerging in this theme. However, this theme also resonated with the confusion regarding the purpose of Playscan. Examples were: “*Why is roulette not working*” and “*Having trouble getting the visa card to transfer money to the playing card*.”

### Quantitative Analyses

**Fig. 1:**
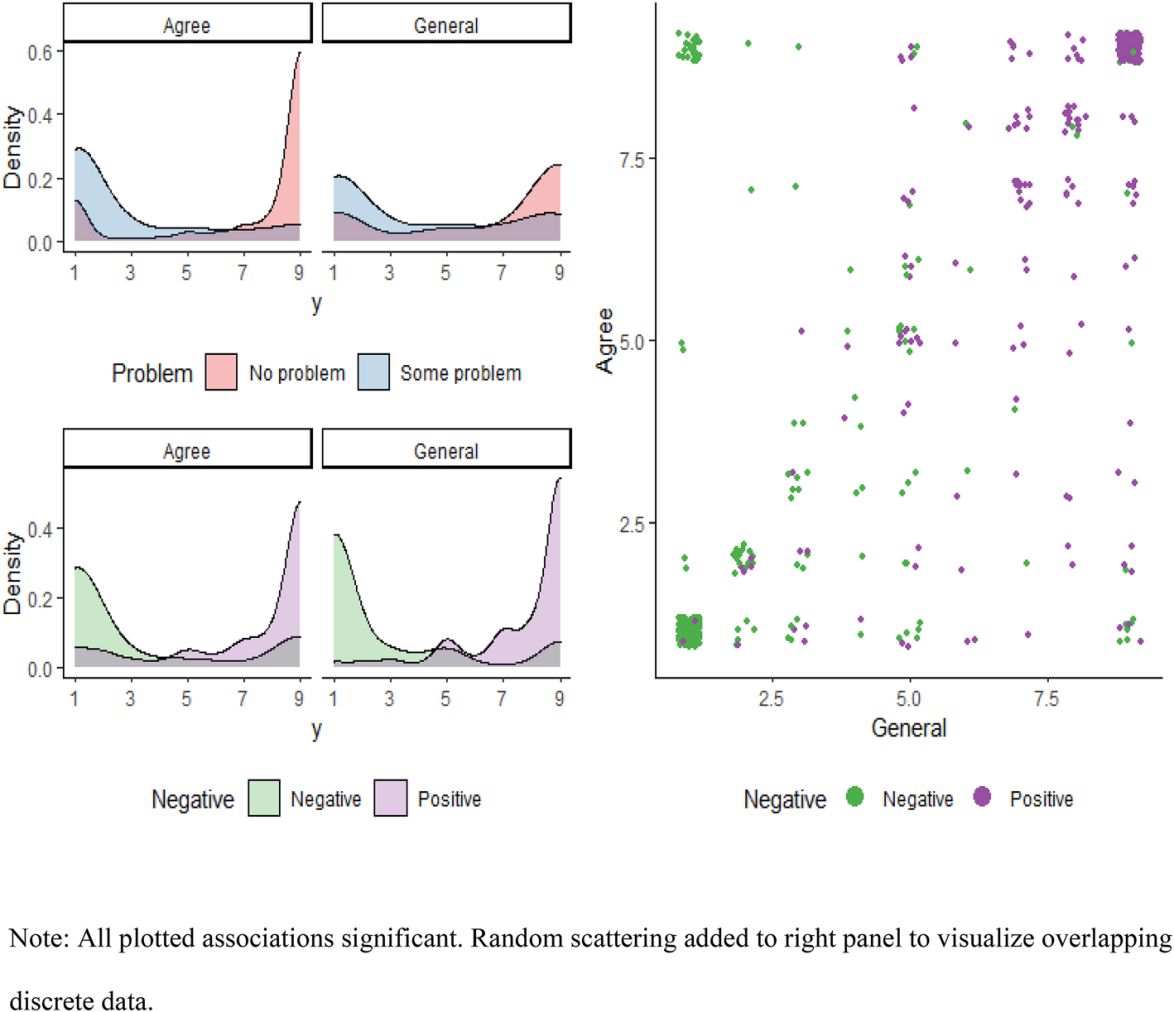
Univariate associations between variables used in multivariate model

Univariate models showed significant associations of expressing a negative theme with assessment agreement (B = − 4.13; 95% CI: − 4.62, − 3.59), and general view of Playscan (B = − 5.02; 95% CI: − 5.38, − 4.57). Furthermore, assessment agreement was also significantly associated with the general view of Playscan (B = 7.32; 95% CI: 0.68, 0.78). Moreover, individuals at a high risk of developing gambling problems displayed significantly lower assessment agreement (B = − 4.36; 95% CI: − 4.79, − 3.85) and general view of Playscan (B = − 2.63; 95% CI: − 3.14, − 2.08). Figure 1 visualizes these findings. Individuals at a high risk of developing gambling problems were significantly more likely to express a negative theme than individuals with a lower risk (odds ratio [OR] = 2.39, *p* < 0.001).

The moderated mediation model revealed that the association between expressing a negative theme and the general view of Playscan was indirectly partially mediated via assessment agreement (B = − 1.74, SE = 0.21, *p* < 0.001), irrespective of the risk of developing gambling problems. A high risk of developing gambling problems did not moderate any paths (see Table 2 for the full results).

**Table 2:** Results of moderated mediation model

Moderator	Type	Effect	Estimate	Lower	Upper	*p*
Average	Indirect	${\text{Expressed negativity}} \Rightarrow {\text{Agree}} \Rightarrow {\text{General}}$	− 1.736	− 2.151	− 1.347	< .00001
Average	Path	${\text{Expressed negativity}} \Rightarrow {\text{Agree}}$	− 3.354	− 3.770	− 2.837	< .00001
Average	Path	${\text{Agree}} \Rightarrow {\text{General}}$	0.158	0.429	0.607	< .00001
Average	Direct	${\text{Expressed negativity}} \Rightarrow {\text{General}}$	− 2.920	− 3.509	− 2.288	< .00001
Average	Total	${\text{Expressed negativity}} \Rightarrow {\text{General}}$	− 4.660	− 5.046	− 4.275	< .00001
Stratified by moderator						
No problem	Indirect	${\text{Expressed negativity}} \Rightarrow {\text{Agree}} \Rightarrow {\text{General}}$	− 1.555	− 2.183	− 1.032	< .00001
No problem	Path	${\text{Expressed negativity}} \Rightarrow {\text{Agree}}$	− 3.255	− 3.929	− 2.580	< .00001
No problem	Path	${\text{Agree}} \Rightarrow {\text{General}}$	0.478	0.348	0.606	< .00001
No problem	Direct	${\text{Expressed negativity}} \Rightarrow {\text{General}}$	− 3.319	− 4.239	− 2.447	< .00001
No problem	Total	${\text{Expressed negativity}} \Rightarrow {\text{General}}$	− 4.874	− 5.403	− 4.345	< .00001
Some problem	Indirect	${\text{Expressed negativity}} \Rightarrow {\text{Agree}} \Rightarrow {\text{General}}$	− 1.926	− 2.631	− 1.377	< .00001
Some problem	Path	${\text{Expressed negativity}} \Rightarrow {\text{Agree}}$	− 3.454	− 4.087	− 2.778	< .00001
Some problem	Path	${\text{Agree}} \Rightarrow {\text{General}}$	0.558	0.414	0.690	< .00001
Some problem	Direct	${\text{Expressed negativity}} \Rightarrow {\text{General}}$	− 2.521	− 3.423	− 1.689	< .00001
Some problem	Total	${\text{Expressed negativity}} \Rightarrow {\text{General}}$	− 4.447	− 5.008	− 3.885	< .00001

## Discussion

### Positive View of the Risk Assessment

Most responses from the users were positive and focused on the beneficial aspects of the risk assessment and RG tool. Users’ positive attitude in the current study, towards the tool and by extension, the received risk assessment, was consistent with Forsström et al’s. (2017) findings. However, the positive comments did not outline any specific information regarding the beneficial aspects of Playscan or its functions and the risk assessment, but discussed generic positive features instead. A tentative conclusion is that the participants lacked the knowledge or interest in discussing the different aspects of the tool in detail. Forsström et al. (2017) reported similar results with participants, who did not discuss the features of the tool. Both study findings may indicate participants’ lack of commitment to the tool. Most participants highlighted another important feature in the comments indicating that the tool did not have any utility for themselves, but could prove useful for other users. In line with the previous findings, this result was consistent with Forsström et al’s. (2017) study. This finding can be understood as a consequence of the “user paradox,” which suggests that the respondents reported a positive view of the overall tool, but did not feel inclined to use it or could not understand the benefits of using it themselves (Forsström et al., 2017). This opinion could be a mechanism to resolve cognitive dissonance, which may have arisen due to the higher-than-expected risk rating received in their feedback, without an inclination to change their attitudes or behavior (Festinger, 1957). However, this is a mere hypothesis that should be tested in future studies.

### Confusion about the Purpose of Playscan and Understanding the Technical Questions

Confusion about the purpose of Playscan was another emergent theme. This theme is consistent with a previous study finding (Forsström et al., 2017). In both these studies, some participants seemed to experience difficulty in differentiating between the tool and gambling website. This indicates that additional information may be required to explain the purpose of Playscan and the function it serves for each player. Thus, this finding could also indicate gamblers’ lack of interest in this type of a tool and its functions. Therefore, providing more information about the RG measures in general could help in resolving the arising confusion.

On the other hand, the comments regarding technical problems on the website might indicate the lack of availability of technical support or information for the users, which may add to the confusion regarding the actual purpose of Playscan on the website. Thus, a direct and transparent way might be needed to present RG measures as independent from the gambling website and to solve technical problems. Lole et al. (2019) used eye-tracking to compare sports bettors’ fixation on RG messages vs wagering-related information, while using a gambling website; the study found that the number of fixations for RG messages was lower than those for wagering-related information, suggesting that the current RG messages displayed on the website were not sufficient to reduce the risks among gamblers. This lower fixation on RG measures has also been reported by several other studies (Forsström et al., 2016, 2017, 2020b; Lole et al., 2019). This conclusion could be extended to the RG messages on all gambling websites, thereby, causing difficulty in differentiating between RG tools and gambling-related material. Therefore, further studies may be required to examine gamblers use and engagement of RG measures.

### Negative View of Playscan and Lower Level of Assessment Agreement

The results of the quantitative analyses revealed that participants at some degree of risk of developing gambling problems reported lower agreement with the risk assessment, lower general assessment of the Playscan, and were more likely to express negative-themed comments. Ivanova et al. (2019) reported consistent findings suggesting that problem gamblers shared negative attitudes toward RG measures in general. The risk assessment is based on the gambling data and acts as an aggregated measure of gambling. Thus, it is not surprising that the individuals receiving a high risk assessment for developing gambling problems rated the tool as less accurate. However, in the current study, the moderated mediation analysis also revealed that the association between expressing a negative theme and general view towards Playscan was partially mediated by assessment agreement, but this mediating path was not moderated by a high risk of developing gambling problems. These analyses show that despite the association between a high risk of developing gambling problems and an overall and specific negative attitude toward RG tools, assessment agreement appears to be the main predictor of general attitude, irrespective of the risk of developing gambling problems. This finding is encouraging because it suggests that no special considerations are necessary prior to implementing RG measures—among individuals with the risk of developing gambling problems—such as self-assessments, which should report high face validity and convey the results in a manner that does not provoke disagreement. Additionally, disagreement regarding the assessment could indicate the underestimation of losses and by extension the degree of risk, by the gamblers. Auer and Griffiths (2017) reported a similar underestimation of losses among gamblers as opposed to their actual gambling data. Thus, these findings have several practical implications to further discuss the patterns of underestimating loss and risk among gamblers, and the lower inclination of problem gamblers to perceive RG measures as something positive (Ivanova et al., 2019). Furthermore, assessment agreement is crucial for reviewing negative themes because several respondents found the assessment nonsensical, and this view would probably determine their future usage of the tool.

### Practical Implications

Previous studies have shown the importance of tailored feedback (Auer et al., 2018; Auer & Griffiths, 2020; 2016; 2018; Gainsbury et al., 2018). The qualitative and quantitative results of our study highlight the need for increased tailored feedback and the incorporation of background information (e.g., income) to ameliorate the assessment.

It is instrumental to devise strategies to deal with lower levels of assessment agreement for improving the usage and overall view of Playscan. The first step would be to provide an opportunity for all users to rate their level of agreement. This agreement score combined with the actual risk assessment may provide empirical support for a high predictive value in machine learning models designed to predict high risk for problem gambling (Deng et al., 2019). Furthermore, follow up questions via chat or email, regarding an individual’s lower assessment agreement, could help in decreasing the negative attitudes toward the assessment received. Such a strategy may transform a negative event into a positive one and increase individual commitment toward the tool.

Additionally, the lack of understanding about the purpose of Playscan indicated a need for making more information available about the tool on the gambling website. Thus, future research should focus on developing a program that educates gamblers about responsible gambling, regardless of the implemented measures.

### Limitations

Our study reported two general limitations of using account-based data for conducting risk assessments of Playscan: a gambler could use several websites for gambling simultaneously and several gamblers could access one account. This may have affected our study findings because the risk assessment functions on a one account-one customer basis; therefore, it remains unknown if the gamblers are aware that the risk assessment is only for one individual. Moreover, no information was available regarding participant’s usage of other gambling websites. This information could have increased our understanding of participants’ perception about their risk assessment.

The current study used an algorithm to determine the level of risk. However, the lack of methods to validate the resulting risk assessment could lead to lower classification accuracy, which may cause lower agreement with a high risk, rather than due to cognitive dissonance.

Existing studies on gambling, particularly those investigating gambling-related problems, are often susceptible to selection bias during participant recruitment. In our study, participation was voluntary, which may affect the sample representativeness of the gambler population using Norsk Tipping’s gambling website, thereby, making it difficult to generalize our findings. However, our findings are informative for understanding gamblers’ behaviors. For instance, few studies investigated the self-exclusion at a later time point among a sample of gamblers that contacted the customer support services at a large gambling company (Haefeli et al., 2011, 2015). Despite the inclusion of a non-representative sample, the study findings made an important contribution to understanding gamblers’ behaviors upon self-exclusion.

Another limitation is the lack of information regarding the degree and duration of Playscan usage. Users may have not had enough experience with Playscan to achieve a comprehensive view of the risk assessment and the overall tool. A study by Forsström et al. (2016) experienced a similar limitation; thus, future studies should conduct more in-depth studies to mitigate this issue. Lastly, self-report measures are inherently limited due to response bias in the data collected. However, this bias can have a greater impact on the results of gambling research because gamblers are prone to act on cognitive biases. Several existing studies have examined cognitive biases and distortions among gamblers (Blaszczynski, 2004; Griffiths, 1994; McCusker & Gettings, 1997). Kuentzel et al. (2008) found that self-reported data for gambling problems may be influenced by individuals’ desire to present an overly positive image of themselves, which would increase the likelihood of underestimating their problems.

### Future Research

Future research should focus on more systematically investigating gamblers’ perception of their risk assessment. Another potential approach is to linking gambling data with individuals who gamble on a regular basis, to understand the individual perception of their gambling, and the association between gambling risk and individual gambling patterns. Furthermore, targeting specific populations such as high-risk gamblers or gamblers focused on a specific activity, for example, casino games could provide an initial understanding of the experience among gamblers that use RG tools. Gamblers’ perception of their own data could also be another interesting method of conceptualizing and examining gamblers’ understanding of their behavior. In addition, investigating the most effective method to present RG information on gambling sites is an important area for future researchers. Thus, there is a need to explore the field of RG tools in detail to investigate some of the contradictory findings present within the research field.

## Conclusions

Our study results indicate some respondents’ disagreements while receiving feedback. Therefore, there is a need for a pedagogical approach when providing feedback to gamblers. In addition, accessibility to more information and assistance in various ways for gamblers is essential for deeper understanding of their level of risk, reducing disagreement, promoting and encouraging further use, and harm minimization.
